# Risk factors for chronic obstructive pulmonary disease (COPD) in a tertiary health institution in Lagos, Nigeria

**DOI:** 10.4314/gmj.v57i3.3

**Published:** 2023-09

**Authors:** Obianuju B Ozoh, Sandra K Dede, Ogochukwu A Ekete, Oluwafemi O Ojo, Michelle G Dania

**Affiliations:** 1 Department of Medicine, Faculty of Clinical Sciences, College of Medicine, University of Lagos, Lagos, Nigeria; 2 Department of Medicine, Lagos University Teaching Hospital, Lagos, Nigeria; 3 Department of Medicine, Lagos State University College of Medicine Ikeja, Lagos, Nigeria

**Keywords:** COPD, Nigeria, risk factors, asthma

## Abstract

**Objective:**

To describe the clinical characteristics and identifiable risk factors for Chronic Obstructive Pulmonary Disease (COPD) in a real-world clinical setting.

**Design:**

Cross-sectional study among patients with COPD.

**Setting:**

The Respiratory clinic of the Lagos University Teaching Hospital.

**Participants:**

Consecutive patients with spirometry confirmed COPD on follow-up for ≥3 months. There were 79 participants.

**Intervention:**

None

**Main outcome measure:**

COPD risk factors, disease severity, comorbidities, and the severity of airflow limitation.

**Results:**

The mean age of the participants was 63.3± 12.4 years, and 47 (59.5) were male. There was a high symptom burden (73.4% had COPD assessment test (CAT) score >10), 33 (41.8%) and 4 (5.1%) had GOLD 3 and GOLD 4 airflow limitation, respectively. Risk factors were identified for 96.2% of the participants: history of asthma in 37 (46.8%), tobacco smoking 22 (27.8%), occupational exposure 15 (19%), biomass exposure 5 (6.6%), post-tuberculosis 3 (3.8%), old age (3.8%), and prematurity 1 (1.3%). Fifty-nine (74.7%) had Asthma COPD Overlap (ACO). There were no significant associations between the risk factors and disease severity. Participants with ACO had lower lung function and a high frequency of allergic rhinitis.

**Conclusion:**

Asthma was the most commonly identifiable risk factor for COPD, underscoring asthma risk reduction and management optimisation as priorities toward COPD burden mitigation. Future studies need to validate these findings and identify the predominant COPD phenotypes in our setting.

**Funding:**

None declared

## Introduction

Chronic Obstructive Pulmonary Disease (COPD) is the third leading cause of death globally, with over 80% of mortality occurring in low- and middle-income countries (LMICs).^1^ The absolute number of persons between 30-79 years old living with COPD is also highest in LMICs, where most cases remain underdiagnosed or misdiagnosed.^2^

Recognised risk factors for COPD include cigarette smoking, occupational and environmental exposures including biomass burning, increasing age, family history of COPD, previous tuberculosis, and a history of asthma or atopy.^2^ The contribution of these risk factors to COPD prevalence varies globally, but tobacco smoking remains a major risk factor.^2^ The impact of indoor air pollution from the use of biomass is projected to be high in LMICs; however, it is confounded by poverty and associated social disadvantages that influence lung development and determine household energy choice.^3^

Although atopy and asthma are considered risk factors for COPD, their role in most epidemiological studies has not been quantified. A recent global burden of COPD iteration recognised asthma and previous tuberculosis (TB) as important risk factors for COPD in LMICs.^2^ Similarly, the Burden of Obstructive Lung Disease (BOLD) study in Nigeria identified asthma, previous TB, and low level of education as independent risk factors for COPD, with no association found with tobacco smoking or exposure to indoor air pollution.^4^ These peculiarities regarding the risk factors for COPD in LMICs are important as they inform the development of context-based interventions toward reducing the disease burden.

Asthma is usually characterised by its variability in clinical presentation and airflow limitation that is largely reversible, either spontaneously or following the administration of bronchodilators. However, long-standing uncontrolled asthma could lead to airway remodelling with persistent symptoms and fixed airflow obstruction characteristic of COPD.^5^ The concept of asthma-COPD overlap (ACO) has been described when features of asthma and COPD coexist. A pragmatic consensus definition of ACO includes a history of asthma or post-bronchodilator change in forced expiratory volume in the first second (FEV1) or forced vital capacity (FVC) of >15% and >400 ml in a person >40 years old with spirometry defined COPD.^6^ The current Global Initiative of Asthma (GINA) guideline now uses the term Asthma + COPD rather than ACO.^7^

There is substantial evidence to support an association between a history of asthma and the development of COPD, particularly when no other risk factor is identifiable.^8^ We hypothesize that the increasing prevalence of asthma and high rates of uncontrolled asthma in Nigeria and other sub-Saharan African (sSA) countries are driving the rising burden of COPD in our settings.^9^,^10^ The aim of this study therefore is to describe the clinical characteristics and identifiable risk factors for COPD among patients attending a tertiary hospital respiratory clinic in Nigeria, and also explore the association between COPD risk factors and clinical characteristics.

## Methods

### Study design and setting

The study was a cross-sectional hospital-based study conducted at the Lagos University Teaching Hospital (LUTH) in Lagos, Nigeria, between 2018 and 2019. LUTH is the largest tertiary hospital in Lagos and runs a weekly airway disease clinic. Lagos is one of the fastest-growing urban cities with a very high population density, heavy traffic congestion, and a high level of ambient air pollution.^11^

### Study participants

Persons with persistent respiratory symptoms and spirometry-confirmed COPD (post-bronchodilator airway obstruction) who were being followed up at the clinic were eligible to participate. COPD was defined based on the Global Initiative on Obstructive Lung Disease (GOLD) criteria as the presence of persistent respiratory symptoms with persistent airway obstruction after bronchodilator.^12^ Consecutive patients who had attended the clinic for a minimum of three months and who had received a prescription for the treatment of COPD were recruited for the study.

### Data collection

Using a standard proforma, interviews and examination of participants' medical records were conducted to obtain socio-demographic data, significant occupational exposures, persistent respiratory symptoms (on most days), comorbidities, history of asthma, family history of asthma, current medications, self-reported adherence to medications and history of exacerbations.

Medication adherence was assessed by asking participants if they used their prescribed maintenance medications most days in the preceding month. Those who responded negatively were considered non-adherent.

More than two episodes of exacerbations requiring an emergency room visit or use of oral steroids in the preceding year or one requiring hospitalization were classified as frequent exacerbations.

The participants also completed the COPD assessment test (CAT) questionnaire. The CAT is a validated eight-item questionnaire that assesses globally the impact of COPD on health status based on the severity of symptoms on a 5-point Likert scale.^13^ It has a maximum score of 40, with higher scores denoting a worse quality of life. Scores >10 were classified as high impact of COPD on life.

Using the GOLD 2022 disease severity classification, participants were classified into either of the four groups A-D based on exacerbation history and CAT score as follows: Group A: less symptom; fewer exacerbations, Group B: More symptoms; fewer exacerbations, Group C: less symptoms; more exacerbations and Group D more symptoms; more exacerbations.^12^

Pre and post-bronchodilator spirometry according to the American Thoracic Society/ European Respiratory Society (ATS/ERS) standards using the Vitalograph® spirometer (Model 6800) was done with quality assurance for good test quality.^14^ The Global Lung Function Initiative (GLI) reference equation for African Americans was used to describe spirometry patterns based on the lower limit of normal (LLN).^15^ Severity of airway obstruction post-bronchodilator was classified using the FEV1% predicted as GOLD 1 - mild: FEV1 ≥80% predicted, GOLD 2 - moderate: 50% ≤ FEV1 <80% predicted, GOLD 3 - severe: 30% ≤ FEV1 <50% predicted, GOLD 4 - very severe: FEV1 <30% predicted.

Significant bronchodilator response was considered an improvement in FEV1 or FVC of >15% and 400 ml. Identifiable risk factors for COPD were classified as follows:

- Tobacco smoking: >10 pack years.
- Indoor biomass exposure: the main cook using biomass as primary cooking fuel for at least one year.
- Occupational exposure to dust, fumes, or chemical for at least one year
- History of previous diagnosis of asthma
- Post tuberculosis: history of previous treatment for tuberculosis.
- Age >70 years with no other identifiable risk factor.

### Data analysis

Descriptive statistics (mean + standard deviation, median, frequencies) were used to summarize numerical and categorical variables. The association between COPD risk factors and selected clinical and spirometry parameters was explored using the appropriate chi-square test, Fisher's exact test, or student's t-test. We categorized patients who reported a history of asthma or had >15% and >400 ml improvement in FEV1 or FVC post-bronchodilator as having ACO consistent with the consensus definition and compared their demographic, clinical and spirometry parameters with those categorized as COPD alone. A p-value <0.05 was considered significant for all associations.

### Ethical considerations

Ethical approval for this study was obtained from the LUTH Health Research Ethics Committee (ADM/DCST/HREC/APP/2174). All participants provided written informed consent.

## Results

There were 79 participants whose ages ranged from 3986 years, with a mean age of 63.3±12.4 years. Their demographic and clinical characteristics are shown in Table 1. Most participants were male and over 50 years old. There was a high symptom burden. Fifty-eight (73.4%) had CAT scores >10; a third were frequent exacerbators. Allergic rhinitis was the most common comorbidity.

**Table 1:** Demographic and clinical characteristics of study participants (N=79)

Characteristic	Frequency n(%)
**Age group (in years)**	
39-50	14 (17.7)
51-60	17 (21.5)
61-70	20 (25.3)
71-80	21 (26.6)
>80	7 (8.9)
**Sex**	
Male	47 (59.5)
Female	32 (40.5)
**BMI in kg/m**^**2**^ **(Mean ± SD)**	
Normal (18-25.9)	34 (43.0)
Overweight (26-29.9)	22 (27.8)
Obese (>30)	23 (29.1)
**Chronic Respiratory symptoms**	
Cough	71 (89.9)
Sputum production	54 (68.4)
Shortness of breath on moderate exertion	70 (88.6)
**CAT score**	
>10	58 (73.4)
<10	21 (26.6)
**Frequent exacerbators**	23 (29.1)
**COPD severity classification**	
Group A	16 (20.3)
Group B	40 (50.6)
Group C	5 (6.3)
Group D	18 (22.5)
**Current medication**	
Oral salbutamol	9(11.4)
Inhaled salbutamol	19 (24.1)
Oral corticosteroid	5 (6.3)
Oral theophylline	4 (5.1)
ICS/LABA	26 (32.9)
Tiotropium	12 (15.2)
Montelukast	12 (15.2)
Antihistamine	13 (16.4)
Nasal steroid	3 (3.8)
**Self-reported adherence to COPD medicines**	43 (54.4)
**Comorbidities**	
Hypertension	30 (38.0)
Diabetes	4 (5.1)
Gastroesophageal reflux disease	10 (12.6)
Allergic rhinitis	34 (43.0)

### Spirometry parameters

Spirometry data for participants is shown in Table 2. The mixed pattern (obstructive with low FVC) was the most predominant pattern both pre and post-bronchodilator. Based on the post-bronchodilator FEV1% predicted for the severity of airflow obstruction, 8 (10.1%) were in GOLD 1, 34 (43.0%) in GOLD 2, 33 (41.8%) in GOLD 3 and 4 (5.1%) in GOLD 4.

**Table 2:** Spirometry parameters among participants

% predicted values	Pre (Mean +SD)	Post (Mean +SD)
FEV1 % predicted	47.8 + 18.2	54.0 +19.0
FVC % predicted	69.0 + 19.3	78.0 + 19.0
FEV1/FVC %	54.5 + 11.7	54.6 +11.8
**Spirometry pattern n (%)**		
Obstructive	20 (25.3)	30 (38.0)
Obstructive with low FVC	48 (60.8)	37 (46.8)
Probable restrictive	5 (6.3)	4 (5.1)
Normal	6 (7.6)	8 (10.1)

Footnote: All the participants with normal pattern post-bronchodilator based on the Lower Limit of Normal criteria used in this study were being managed for COPD and had persistent symptoms and FEV1/FVC ratio <70%.

### Identifiable risk factors and association with disease severity

There was an identifiable risk factor for COPD in 76 (96.2%) participants, as shown in Table 3. A history of asthma was the most frequently identified risk factor in 37 (46.8%) participants. Only one participant was a current smoker. About half of the occupational exposures were associated with construction work. Ten (12.7%) participants had two identifiable risk factors, and tobacco smoking was an additional risk in 8 (10.1%).

**Table 3:** Frequency of COPD risk factors

Risk factor	Frequency (%)
History of asthma	37 (46.8)
Tobacco smoking	22 (27.8)
Occupational exposure	15 (19)
Biomass smoke exposure	5 (6.3)
Post tuberculosis	3 (3.8)
Old age	3 (3.8)
Prematurity	1 (1.3)

Only two patients with a history of asthma had smoked tobacco, and none had occupational or biomass exposure. Fifty-six (70.9%) participants reported a family history of asthma. There was no association between specific COPD risk factors and disease severity or degree of airflow obstruction (Table 4).

**Table 4:** Association between identifiable COPD risk factors and disease severity

	History of asthma	Tobacco smoking	Occupational exposure	Biomass exposure	Others	No risk factor	p-value
**The severity of airway obstruction (%)**							
GOLD 1	2 (5.4)	1 (5.0)	1 (12.5)	2 (40.0)	2 (33.3)	0	0.50
GOLD 2	17 (45.9)	10 (50.0)	4 (50.0)	1 (20.0)	1 (16.7)	1 (33.3)	
GOLD 3	16 (43.2)	8 (40.0)	2 (25.0)	2 (40.0)	3 (50.0)	2 (66.7)	
GOLD 4	2 (5.4)	1 (5.0)	1 (12.5)	0	0	0	
**Frequent exacerbator (%)**	13 (35.1)	6 (30.0)	2 (25.0)	0	1 (16.7)	1 (33.3)	0.75
**COPD severity (%)**							
Group A	7 (18.9)	3 (15.0)	1 (12.5)	2 (40.0)	2 (33.3)	1 (33.3)	0.90
Group B	17 (45.9)	11 (55.0)	5 (62.5)	3 (60.0)	3 (50.0)	1 (33.3)	
Group C	2 (5.4)	3 (15.0)	0	0	0	0	
Group D	11 (29.7)	3 (15.0)	2 (25.0)	0	1 (16.7)	1 (33.3)	

Footnote: Where there were two risk factors, e.g. tobacco smoking and occupational exposure, tobacco smoking was chosen.

### Comparison of clinical characteristics and spirometry measures between participants with Asthma+COPD and those with COPD only

The mean percentage post-bronchodilator response was 15.9% ± 18.9 for the FVC and 15.8% ± 19.6 for the FEV1. Forty-four (55.7%) had significant (>15% and 400 ml) post-bronchodilator response. Half of these did not report a history of asthma.

A total of 59 (74.7%) participants met the criteria for ACO (>15% post-bronchodilator response or a history of asthma). A comparison of the clinical and spirometry parameters between those with ACO and COPD alone is shown in Table 5. There was no difference in clinical characteristics between those with ACO and those with COPD alone. The mean pre-bronchodilator FEV1 and FVC were significantly lower among those with ACO. Tobacco smoking was significantly higher among those with COPD alone.

**Table 5:** Comparison of clinical and spirometry parameters between Asthma+COPD and COPD alone

Variables	Asthma+COPD (n=59)	COPD alone (n=22)	p-value
**Age group in years (%)**			0.70
**39-50**	12 (20.3)	2 (10.0)	
**51-60**	12 (20.3)	5 (25.0)	
**61-70**	15 (25.4)	5 (25.0)	
**71-80**	16 (27.1)	5 (25.0)	
**>80**	4 (6.8)	3 (15.0)	
**Sex (%)**			0.27
**Male**	33 (55.9)	14 (70.0)	
**Female**	26 (44.1)	6 (30.0)	
**BMI (%)**			0.70
**Normal (18-25.9)**	26 (44.1)	8 (40.0)	
**Overweight (26-29.9)**	15 (25.4)	7 (35.0)	
**Obese (>30)**	18 (30.5)	5 (25.0)	
**Chronic Respiratory symptoms (%)**			
**Cough**	53 (89.8)	18 (90.0)	0.98
**Sputum production**	40 (67.8)	14 (70.0)	0.86
**Shortness of breath on moderate exertion**	53 (89.8)	17 (85.0)	0.56
**Allergic rhinitis (%)**	29 (49.2)	5 (25.0)	0.06
**CAT score >10 (%)**	42 (71.2)	16 (80.0)	0.44
**Frequent exacerbator (%)**	19 (32.2)	4 (20.0)	0.30
**COPD severity classification (%)**			0.12
**Group A**	14 (23.7)	2 (10.0)	
**Group B**	24 (44.1)	14 (70.0)	
**Group C**	3 (5.1)	2 (10.0)	
**Group D**	16 (27.1)	2 (10.0)	
**Pre-FEV1 %predicted in liters (Mean+SD)**	44.0 (16.4)	58.9 (19.0)	0.001
**Pre-FVC %predicted (Mean+SD)**	64.9 (17.7)	80.8 (19.4)	0.001
**Post FEV1 %predicted (Mean+SD)**	51.6 (18.2)	61.0 (19.8)	0.06
**Post FVC %predicted**	76.2 (18.7)	83.6 (19.3)	0.13
**Severity of airflow obstruction**			0.46
**GOLD 1**	5 (8.5)	3 (15.0)	
**GOLD 2**	24 (40.7)	10 (50.0)	
**GOLD 3**	26 (44.1)	7 (35.0)	
**GOLD 4**	4 (6.8)	0	
**Tobacco smoking (%)**	12 (20.3)	10 (50.0)	0.02
**Occupational exposure (%0**	9 (15.3)	6 (30.0)	0.15

## Discussion

Among these patients attending a tertiary care hospital for COPD treatment, there is a very high symptom burden, moderate to severe airflow limitation and high rates of comorbidities. A history of asthma was the most frequently identified risk factor for COPD, accounting for nearly half of all risk factors. The severity of airflow limitation and clinical characteristics were similar across COPD risk factors. Three-quarters met the definition for Asthma+COPD or ACO and these had significantly lower pre-bronchodilator FEV1 and compared to the group with COPD alone.

The contribution of asthma as a risk factor for COPD appears more prevalent in LMIC, as shown in previous epidemiologic studies and supported by this real-life study.^2^,^4^ Longitudinal studies and systematic reviews have also provided evidence to support the hypothesis that asthma is a common risk factor for COPD.^8^,^16^,^17^ In the longitudinal study by Silva et al., participants with asthma compared to those without asthma had a 10 times higher risk for incident diagnosis of chronic bronchitis, 17 times higher risk for emphysema, and 12.5 times higher risk for COPD after adjusting for smoking history and other potential confounders.^17^ Furthermore, fixed airway obstruction meeting the spirometry definition for COPD has been reported in about 50% of cases of asthma, being worse in severe and uncontrolled asthma.^18^,^19^

Similar to findings from previous studies, persons with COPD and a history of asthma as the risk factor in this study had similarities with COPD from other risk factors with regards to disease severity.^20^,^21^

Although persons with ACO did not differ from those with COPD alone regarding disease severity or demographics, they had significantly lower pre-bronchodilator lung function with a higher tendency towards having allergic rhinitis and more severe airflow limitation post-bronchodilator. There are reported inconsistencies regarding the rate of lung function decline in ACO compared to COPD alone. Recent studies support a more favourable rate of lung function decline for ACO compared to COPD alone.^22^,^23^ However, this study does not address rate of lung function decline despite suggesting more severe airflow limitation among those with ACO.

Differences between ACO and COPD alone have been recognized. There is usually a higher level of eosinophils in peripheral blood and airways, higher CD4+/CD8+ T cells in the airway mucosa, thicker epithelial basement membrane, higher diffusing capacity, higher exhaled nitric oxide, lower high-resolution computed tomography scan emphysema score, and greater reversibility to bronchodilator in ACO.^21^ There is also a greater response to treatment with steroids in ACO which makes it pertinent to identify patients with ACO for early inclusion of inhaled steroids (ICS) to the bronchodilator treatment.^24^,^7^,^25^

The findings in this present study may suggest an association between the clinical presence of allergic rhinitis and ACO, but this must be substantiated. In the current era in which disease phenotyping and personalized care are priorities for patient care, clinical characteristics and biomarkers are increasingly used to categorize patients. Identifying COPD phenotypes with treatable traits such as Asthma+COPD, frequent exacerbator, eosinophilic or COPD with bronchiectasis guides therapy and informs policy regarding procurement of diagnostic facilities and medicines especially in our resource limited settings. For example ICS is very effective in reducing the frequency of exacerbations when peripheral blood eosinophil count is ≥ 300 cells/µL and identifying the clinical characteristics, including risk factors for this disease phenotype is important.^12^ High burden of ACO in this study may infer that the eosinophilic phenotype predominates our practice. However, future studies that measure peripheral blood eosinophil count will provide underpinning evidence to verify this.

One of the proposed explanations for the co-existence of asthma and COPD is that of a common origin, with environmental and genetic modulations in disease expression. It is postulated that early life exposures such as maternal smoking, indoor air pollution, and social disadvantage factors are associated with the development of ACO, while later life exposures to environmental factors such as tobacco smoke and occupational exposures predict COPD only.^26^ This postulation is plausible and juxtaposes to the findings in this present study in a low-income setting with high rates of poverty and social disadvantages.^27^ Another postulation is that uncontrolled asthma with persistent airway inflammation could lead to lung remodelling, collagen deposition, and fibrosis that dovetails to fixed airway obstruction and COPD 19,28. Again, the high rates of uncontrolled asthma in Nigeria, makes this a plausible explanation for the predominance of a previous diagnosis of asthma among the participants in this study.^10^ It is, therefore, reasonable to posit that mitigating the burden of COPD in the local practice settings hinges on optimal asthma management to achieve asthma control^10^ It also underscores the need to improve the social determinants of health, limit early life exposures to adverse conditions for lung growth, such as air pollution, and enhance access to healthcare for the management of chronic respiratory diseases in sSA.

Moreover, the contribution of asthma to the risk of COPD may be higher than reported in this study, considering the high frequency of allergic rhinitis (43%), family history of asthma (75%,) and proportion with significant (>15%) postbronchodilator response (57.7%). However, it is also known that airway hyperresponsiveness and significant bronchodilator response can occur in COPD without a previous diagnosis of asthma, as also seen in this present study, where half of those with significant bronchodilator response did not report a previous diagnosis of asthma.^25^ In Nigeria, however, under-diagnosis of asthma is also very common, and the reliance on self-reported or documented diagnosis of asthma may have led to the underestimation of the burden of asthma among these patients.^29^

Risk factors for COPD in clinical settings in sSA have not been extensively reported. In the study by Ojuawo et al in a tertiary clinical setting such as ours, patients with a history of asthma were excluded, making their findings incomparable with the present study.^30^ However, they reported that non-tobacco-related exposures were the predominant risk factors (57.6%), with exposure to biomass being the main risk factor.^30^ In contrast, tobacco smoking was the second-ranking risk factor for COPD, higher than biomass and occupational exposures in this present study. Strengthening tobacco control measures remains a public health priority for reducing the burden of COPD, including implementation of regulations that limit occupational exposures.^31^

The sample size and the single-centre experience reported in this study are limitations to the generalizability of the findings to other clinical settings. Also, given the cross-sectional study design, no causal inference can be drawn from the data provided. Nonetheless, this study will drive future prospective studies aimed at COPD phenotyping in sSA.

## Conclusion

Among patients with COPD attending a tertiary care hospital, a history of asthma was the most common risk factor for COPD. There was a high burden of ACO in three-quarters of the participants. Tobacco smoking was also an important risk factor for COPD. These findings underscore asthma risk reduction and management optimisation as priorities toward COPD burden mitigation. Future studies are recommended to validate these findings and identify the predominant COPD phenotypes in other practice settings.
